# Molecular detection of *Anaplasma* infections in ixodid ticks from the Qinghai-Tibet Plateau

**DOI:** 10.1186/s40249-019-0522-z

**Published:** 2019-02-07

**Authors:** Rong Han, Ji-Fei Yang, Muhammad Uzair Mukhtar, Ze Chen, Qing-Li Niu, Yuan-Qing Lin, Guang-Yuan Liu, Jian-Xun Luo, Hong Yin, Zhi-Jie Liu

**Affiliations:** 1 State Key Laboratory of Veterinary Etiological Biology, Key Laboratory of Veterinary Parasitology of Gansu Province, Lanzhou Veterinary Research Institute, Chinese Academy of Agricultural Sciences, Xujiaping 1, Lanzhou, 730046 China; 2 Qinghai Provincial Center for Animal Disease Control and Prevention, Xining, 810003 China; 3 Jiangsu Co-innovation Center for Prevention and Control of Important Animal Infectious Diseases and Zoonoses, Yangzhou, 225009 China

**Keywords:** *Anaplasma*, Tick, Sequence analysis, Prevalence

## Abstract

*Anaplasma* species are tick-transmitted obligate intracellular bacteria that infect many wild and domestic animals and humans. The prevalence of *Anaplasma* spp. in ixodid ticks of Qinghai Province is poorly understood. In this study, a total of 1104 questing adult ticks were investigated for the infection of *Anaplasma* species. As a result, we demonstrated the total infection rates of 3.1, 11.1, 5.6, and 4.5% for *A. phagocytophilum*, *A. bovis*, *A. ovis* and *A. capra*, respectively. All of the tick samples were negative for *A. marginale*. The positive rates of *A. phagocytophilum*, *A. ovis* and *A. capra* in different tick species were significantly different. The positive rates of *A. capra* and *A. bovis* in the male ticks were significantly higher than that in the female ticks. Sequence analysis of *A. ovis* showed 99.5–100% identity to the previous reported isolates. The sequences of *A. phagocytophilum* had 100% identity to strains Ap-SHX21, JC3–3 and ZAM dog-181 from sheep, Mongolian gazelles, and dogs. Two genotypes of *A. capra* were found based on 16S rRNA, citrate synthase (*gltA*) gene and heat shock protein (*groEL*) gene analysis. In conclusion, *A. bovis*, *A. ovis*, *A. phagocytophilum*, and *A. capra* were present in the ticks in Qinghai Province. *Anaplasma* infection is associated with tick species, gender and distribution. These data will be helpful for understanding prevalence status of *Anaplasma* infections in ticks in Qinghai-Tibet Plateau.

**Electronic supplementary material:**

The online version of this article (10.1186/s40249-019-0522-z) contains supplementary material, which is available to authorized users.

## Multilingual abstracts

Please see Additional file 1 for translations of the abstract into the five official working languages of the United Nations.

## Background

Ticks are important vectors of many viral, bacterial, and protozoal pathogens that infect to humans and animals, and tick species are widely distributed all over the world. Among tick-borne pathogens, the genus *Anaplasma* (order Rickettsiales, family Anaplasmataceae) is composed of tick-transmitted obligate intracellular bacteria, which include *A. ovis*, *A. bovis*, *A. marginale*, *A. phagocytophilum*, *A. platys*, *A. centrale* and *A. capra* [1, 2]. *A. ovis* is an obligate intra-erythrocytic organism of small ruminants. *A. centrale* has relatively mild virulence and it has been used as a live vaccine against *A. marginale* infection in several countries [3]. *A. bovis* infects monocytes of small mammals and ruminants [4, 5]. *A. phagocytophilum* infects neutrophils of many wild and domestic animals and humans, is the etiological agent of human granulocytic anaplasmosis and tick-borne fever [6]. *A. platys* is unique in infecting the platelets of dogs and it is the etiological agent of the infectious canine cyclic thrombocytopenia [7]. *A. capra* has been identified in China as a novel tick-transmitted zoonotic pathogen but its vectors and infected cell types are unclear [1]. Ixodid ticks play a critical role in the transmission and maintenance of *Anaplasma* species [8]. *Dermacentor nuttalli*, *Hyalomma asiaticum* and *Rhipicephalus pumilio* are the main vectors of *A. ovis* in China [9]. Although ixodid tick infestation of livestock is common, little is known about the *Anaplasma* infection in the ticks in Qinghai Province.

Qinghai Province is located in the northeastern part of Qinghai-Tibet Plateau in western China. Qinghai has an average attitude of more than 3000 m with 54% of the total area being between 4000 m and 5000 m. The provincial climate is characterized by being relatively arid, windy, and cold. Qinghai contains significant amounts of pastures and is an important region for animal production. Qinghai has 33.45 million ha of grassland. The grassland meadows are classified as alpine, swamp, Gobi, forest, and prairie. Yaks, Tibetan sheep, sheep and goats are adapted for survival and growth on these grasslands. Ixodid ticks infestation of livestock is often found in Qinghai Province, including 54.5, 24.0, 36.1% infection rates of *A. ovis* in sheep [10], *Babesia* spp. in wild yaks [11], and *Theileria* spp. in yaks [12], respectively. However, very little is known about the *Anaplasma* infection in animals and ticks. In this study, we identified and analyzed the infections of *A. phagocytophilum*, *A. bovis*, *A. ovis*, *A. marginale* and *A. capra* in ticks. The data provide an overview of *Anaplasma* infections and the potential threats to both livestock and humans in the study areas.

## Methods

### Sampling sites and tick collection

Samples were collected in the Qinghai Province, the Qinghai-Tibetan Plateau at an average altitude of > 3000 m. From February to October in 2015–2017, a total of 1104 questing adult ticks were collected from vegetation on 22 counties of Qinghai by using the flagging method. All of the tick specimens were identified according to morphological criteria [13] and a few were confirmed by sequence analysis of a partial fragment of the 16S rRNA gene.

### DNA extraction, PCR amplification and sequencing

DNA extraction of each individual ticks was conducted as described previously [2]. DNA samples were detected for the presence of the agents in the genus *Anaplasma* by PCR targeting the *msp*4 gene for *A. ovis* and *A. marginale*, the 16S rRNA gene for *A. phagocytophilum* and *A. bovis*, and the citrate synthase (*gltA*) gene for *A. capra*, respectively. For further confirmation of the *A. capra*, the 16S rRNA gene and the heat-shock protein gene (*groEL*) were amplified from *A. capra* positive samples. The 16S rRNA gene was amplified for the molecular identification of the tick species. The PCR was carried out by using an automatic thermocycler (Bio-Rad, Hercules, USA). The reaction system for the PCRs was the same as described in our previous study [14] and the PCR primers and cycling conditions were shown in Table 1. The DNAs extracted from the animals infected with *A. ovis*, *A. marginale*, *A. phagocytophilum*, *A. bovis* and *A. capra* were used as positive controls, and double distilled water was used as a negative controls. The PCR products were visualized under UV illumination in a 1.2% agarose gel followed by electrophoresis and treated with GoldView I (Solarbio, Beijing, China).

**Table 1:** Primers used for PCR for the identification of tick species and detection and of *Anaplasma* spp. in the ticks from Qinghai

Target species	Target gene	Primer(5′ → 3′)	Annealing temperature (°C)	No. of cycles	Expected size (bp)	References
*Anaplasma* spp.	16S rRNA	EE1: TCCTGGCTCAGAACGAACGCTGGCGGC EE2: AGTCACTGACCCAACCTTAAATGGCTG	55	35	1400	[37]
*A. bovis*	16S rRNA	AB1f: CTCGTAGCTTGCTATGAGAAC AB1r: TCTCCCGGACTCCAGTCTG	55	35	551	[26]
*A. phagocytophilum*	16S rRNA	SSAP2f: GCTGAATGTGGGGATAATTTAT SSAP2r: ATGGCTGCTTCCTTTCGGTTA	55	35	641	[26]
*A. marginale*	*msp*4	Amargmsp4F: CTGAAGGGGGAGTAATGGG Amargmsp4R: GGTAATAGCTGCCAGAGATTC	60	30	344	[38]
*A. ovis*	*msp*4	MSP43: CCGGATCCTTAGCTGAACAGAATCTTGC MSP45: GGGAGCTCCTATGAATTACAGAGAATTGTTTAC	60	35	869	[39]
*A. capra*	*gltA*	gltAouterF: GCGATTTTAGAGTGYGGAGATTG gltAouterR: TACAATACCGGAGTAAAAGTCA	55	35	1031	[1]
		gltAinnerF: GCGATTTTAGAGTGYGGAGATTG gltAinnerR: GCGATTTTAGAGTGYGGAGATTG	60	35	594	
	16S rRNA	Forward: GCAAGTCGAACGGACCAAATCTGT Reverse: CCACGATTACTAGCGATTCCGACTTC	58	35	1261	[35]
	*groEL*	Forward: TGAAGAGCATCAAACCCGAAG Reverse: CTGCTCGTGATGCTATCGG	55	35	874	[35]
Tick	16S rRNA	16SrRNA-F: CTGCTCAATGATTTTTTAAATTGCTGTGG 16SrRNA-R: CCGGTCTGAACTCAGATCAAGT	55	35	450	Designed for this study

The PCR products were purified with the TaKaRa Agarose Gel DNA Purification Kit Ver.2.0 (TaKaRa, Dalian, China). Purified PCR products were cloned into a pGEM-T Easy vector (Promega, Madison, WI, USA), and then transformed into *Escherichia coli* JM109 competent cells (TaKaRa, Dalian, China). Three positive colonies from each sample were subjected to sequencing. The obtained sequences were used to conduct BLAST search in GenBank® of the National Center for Biotechnology Information (https://blast.ncbi.nlm.nih.gov/Blast.cgi).

### Data analysis

The data were grouped into three variables in terms of tick species, tick gender and the altitude of the sampling sites, respectively. Differences in each group were statistically calculated using a Chi-square test in Predictive for Analytics Software Statistics 18 (PASW, SPSS Inc.,Chicago, IL, USA). A *P*-value of < 0.05 was considered significant.

## Results

### Identification of the tick species

**Fig. 1:**
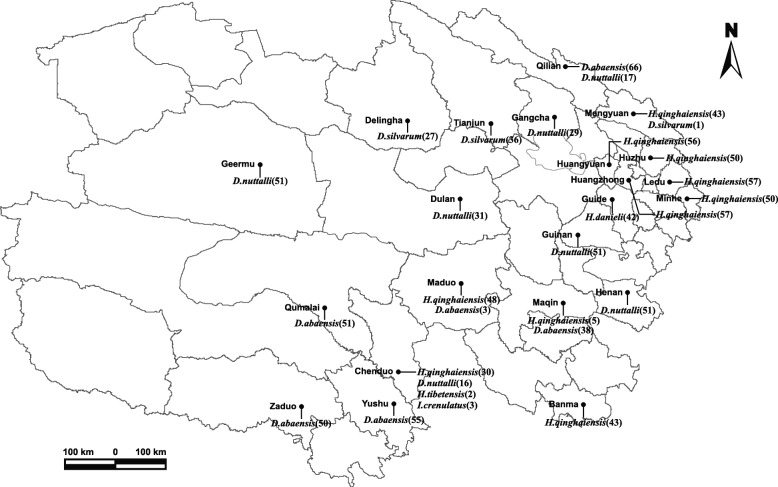
Map of the Sampling sites and the distribution of the collected tick species in Qinghai Province

A total of 1104 questing adult ticks (512 female, 592 male) were collected from vegetation in 22 counties of Qinghai Province. The ticks included seven species in three genera. There were 454 *Haemaphysalis qinghaiensis*, 263 *D. abaensis*, 246 *D. nuttalli*, 94 *D. silvarum*, 42 *H. danieli*, 3 *Ixode crenulatus* and two *H. tibetensis* respectively (Fig. 1). The species of ticks identified by morphology and supported by sequence analysis. The 16S rRNA sequence of *H. qinghaiensis* showed 100% identity to *H. qinghaiensis* isolate HY21 (GenBank accession number: MF629877) from Huangyuan in Qinghai; *D. nuttalli* and *D. silvarum* showed 99% similarity to *D. nuttalli* isolate HBS5(GenBank accession number: KU558731) and *D. silvarum* isolate Hebei (GenBank accession number: JF979379) from Hebei Province in China. The 16S rRNA sequences of *H. danieli*, *H. tibetensis*, *D. abaensis* and *I. crenulatus* were obtained for the first time.

### Detection of the *Anaplasma* spp. in ticks

Five *Anaplasma* species were investigated in the ticks. Of the 1104 samples tested, the average infection rates were 3.1, 11.1, 5.6, and 4.5% for *A. phagocytophilum*, *A. bovis*, *A. ovis*, and *A. capra*, respectively. All of the samples were negative for *A. marginale*. *A. phagocytophilum* was detected in four tick species from ten sampling sites, and it was detected for the first time in *D. abaensis*, *D. nuttalli,* and *H. danieli*. *A. bovis* was detected in five tick species from 14 sampling sites, whereas *A. ovis* was detected in three tick species from nine sampling sites. Three tick species including *H. qinghaiensis*, *D. abaensis* and *D. nuttalli* were infected by *A. capra*. The prevalence of *Anaplasma* spp. in each sampling site is shown in Table 2.

**Table 2:** Detection of *Anaplasma* spp. in the ticks collected from 22 counties in Qinghai Province

County/Average altitude	Tick species	Number of tested	Number of infected (*n*)/Infection rate (%)			
			*A. phagocytophilum*	*A. bovis*	*A. ovis*	*A. capra*
Ledu/2000 m	*H. qinghaiensis*	57	0	0	10/17.5	17/29.8
Huangzhong/2645 m	*H. qinghaiensis*	57	7/12.3	14/24.6	5/8.8	14/24.6
Qumalai/4223 m	*D. abaensis*	51	0	2/3.9	0	5/9.8
Yushu/4493 m	*D. abaensis*	55	0	16/29.1	0	7/12.7
Maduo/4300 m	*H. qinghaiensis*	48	1/2.1	9/18.8	0	1/2.1
	*D. abaensis*	3	0	0	0	0
Maqin/3730 m	*D. abaensis*	38	2/5.3	7/18.6	3/7.9	1/2.6
	*H. qinghaiensis*	5	0	1/33.3	0	0
Mengyuan/2880 m	*H. qinghaiensis*	58	1/1.7	19/32.8	0	0
	*D. silvarum*	1	0	0	0	0
Tianjun/3180 m	*D. silvarum*	54	0	0	0	0
Delingha/2980 m	*D. silvarum*	39	0	0	0	0
Chengduo/4500 m	*D. nuttalli*	16	0	0	0	2/12.5
	*H. qinghaiensis*	30	5/16.7	4/15.2	0	0
	*H. tibetensis*	2	0	0	0	0
	*I. crenulatus*	3	0	1/33.3	0	0
Banma/3560 m	*H. qinghaiensis*	43	0	0	0	0
Gangcha/3300 m	*D. nuttalli*	29	0	0	0	0
Huangyuan/2666 m	*H. qinghaiensis*	56	8/14.3	11/19.6	0	1/1.8
Qilian/2810 m	*D. abaensis*	66	0	2/3.0	6/9.1	0
	*D. nuttalli*	17	0	0	0	0
Dulan/3180 m	*D. nuttalli*	31	1/3.2	0	4/12.9	0
Guinan/3100 m	*D. nuttalli*	51	0	1/2.0	14/27.5	0
Huzhu/2520 m	*H. qinghaiensis*	50	0	5/10.0	0	0
Zaduo/4200 m	*D. abaensis*	50	4/8.0	5/10.0	0	0
Guide/2200 m	*H. danieli*	42	4/8.5	5/100	0	0
Henanxian/3600 m	*D. nuttalli*	51	1/2.0	20/39.2	11/21.6	2/3.9
Minhe/1650 m	*H. qinghaiensis*	50	0	0	3/6.0	0
Geermu/2800 m	*D. nuttalli*	51	0	0	6/11.8	0
Total		1104	34/3.1	122/11.1	62/5.6	50/4.5

Molecular characterization was based on the partial sequences of 16S rRNA gene (642 and 551 bp) for *A. phagocytophilum* and *A. bovis*, *msp*4 gene (869 bp) for *A. ovis*, 16S rRNA, *gltA* and *groEL* genes (1261 bp, 594 bp and 874 bp) for *A. capra*. These sequences were generated from positive samples representing the different sampling sites. As listed in Table 3, *A. ovis* were grouped into four genotypes. *A. phagocytophilum* were classified into three genotypes, and they were 100% identical to sequences of strains Ap-SHX21, JC3–3, and ZAM dog-181 from sheep, Mongolian gazelles, and dogs, respectively. *A. bovis* were classified into five genotypes. The 16S rRNA gene sequences of *A. capra* showed 99.8–100% similarity to strain S62b from sheep and strain 9-13a from goat, and the *groEL* gene sequences were identical with strain tick102/China/2013 and M141a, respectively. These sequences showed a close relation to the sequences of strain HLJ-14 from a patient. In addition, two genotypes of *gltA* gene sequences of *A. capra* were obtained in this study.

**Table 3:** Genotyping of *Anaplasma* spp. in the ticks in Qinghai Province

*Anaplasma* spp.	Gene marker	Number of obtained sequences	Number of genotypes	GenBank accession numbers of obtained sequences	Reference sequences from GenBank
*A. ovis*	16S rRNA	47	4	MG940865, MG940866, MG940868, MG940867	MF071305, HQ456347, EF067341, HQ456350
*A. phagocytophilum*	16S rRNA	56	3	MG940877, MG940878, MG940879	KU321304, KM186948, LC269823
*A. bovis*	16S rRNA	40	5	MG940884, MG940881, MG940880, MG940882, MG940883	KU509990, HQ913645, EU682764, KJ639885, KF465981
*A. capra*	16S rRNA	28	2	MG940874, MG940873	MF066917 KX417196
	*groEL*	20	2	MG940875, MG940876	KR261634, KX685888
	*gltA*	18	2	MG940871 MG940872	KX417308, KX685885

### Risk factors for *Anaplasma* infection to in the tick species

Risk factors, including tick species, gender, and altitude of sampling sites, were used as variables for statistical analysis of the infection patterns of *Anaplasma* spp. (Table 4). As a result, tick species was positively associated with the presence of *A. phagocytophilum*, *A. capra*, and *A. ovis*. *H. danieli* had a higher risk than other tick species to be infected with *A. phagocytophilum*. *D. nuttalli* had a higher risk to be infected with *A. ovis*. *H. qinghaiensis* was most likely to be infected by *A. capra*. Male ticks were more likely to be infected by *A. bovis* or *A. capra* than female ticks. Altitude was a risk factor to *A. phagocytophilum*, *A. bovis* and *A. capra* infections. Ticks collected below 3000 m areas had a higher risk for being infected by *A. phagocytophilum* and *A. capra* than in the ticks collected at elevations greater than 3000 m. *A. bovis* infection rates in ticks collected above 4000 m were higher than in the ticks collected below 4000 m.

**Table 4:** Patterns of *Anaplasma* spp. prevalence in the ticks, grouped by tick species, tick gender and the altitude of the sampling sites

Group		Number of tested	Number of infected (n)/Infection rate (%)							
			*A. phagocytophilum*	*P-value*	*A. bovis*	*P-value*	*A. ovis*	*P-value*	*A. capra*	*P-value*
Tick	*H. qinghaiensis*	454	22/4.8	0.0032	63/13.9	0.230	18/4.0	0.000057	33/7.3	0.0056
	*H. tibetensis*	2	0		0		0		0	
	*H. danieli*	42	4/9.5		5/11.9		0		0	
	*D. abaensis*	263	6/2.3		32/12.2		9/3.4		13/4.9	
	*D. silvarum*	94	0		0		0		0	
	*D. nuttalli*	246	2/0.8		21/8.5		35/14.2		4/1.6	
	*I. crenulatus*	3	0		1/33.3		0		0	
Gender	Female	512	16/3.1	0.935	47/9.2	0.045	23/4.5	0.312	14/2.7	0.0077
	Male	592	18/3.0		75/12.7		39/6.6		36/6.1	
Altitude	≤ 3000 m	461	20/4.3	0.015	54/11.7	0.037	30/6.5	0.316	32/6.9	0.000066
	3000–3900 m	385	4/1.0		31/8.1		32/8.3		3/0.8	
	≥ 4000 m	258	10/3.9		37/14.3		0		15/5.8	

## Discussion

Qinghai is one of the five largest animal grazing regions in China. Grazing animal production is a supporting industry in this region. The Qinghai ecosystem is very suitable for ixodid tick infestation and 25 tick species in six genera has been reported [15]. In this study we collected seven tick species from three genera. These were *H. qinghaiensis*, *H. tibetensis*, *H. danieli*, *D. abaensis*, *D. nuttalli*, *D. silvarum*, and *I. crenulatus*. *H. qinghaiensis* is common in northwestern China, and it has been the dominant tick species in Qinghai since it was initially discovered in Huangyuan County [13]. In the present study, 41.1% of the collected ticks were *H. qinghaiensis*. Three *Dermacentor* spp. ticks (*D. abaensis*, *D. nuttalli* and *D. silvarum*) were frequently encountered on grazing livestock in high altitude areas (2800 to 4300 m), whereas *I. crenulatus* and *H. tibetensis* were rare. To verify the morphological identification of the tick species, the 16S rRNA gene sequences were analyzed. The sequences from *H. qinghaiensis*, *D. nuttalli*, and *D. silvarum* ticks were identical to their corresponding reference sequences in Genbank. The sequences of *H. danieli*, *H. tibetensis*, *D. abaensis* and *I. crenulatus* were compared with our reference sequences (data unpublished) because of the lack of the reference sequences in GenBank.

*Aanaplasma* prevalence in ticks demonstrated a wide distribution of *A. phagocytophilum*, *A. bovis*, *A. ovis* and *A. capra*. Among the *Anaplasma* species, *A. phagocytophilum* is an emerging tick-borne zoonotic pathogen of public health significance [16], and it has been detected in many tick species, including *H. qinghaiensis*, *H. concinna*, *H. longicornis*, *I. persulcatus*, and *D. silvarum* in China [17–20]. We detected *A. phagocytophilum* in *H. qinghaiensis*, and, for the first time, found it in *D. abaensis*, *D. nuttalli*, and *H. danieli*. The 16S rRNA gene sequences represented three genotypes, which showed high identities to the sequences found in goats from Central and Southern China [21], these genotypes were different from the genotype identified from human samples. Therefore, the significance of these genotypes to public health needs further investigation. *A. bovis* was initially found as a pathogen of cattle but has also been reported in sheep, goats, wild deer, and dogs [5, 22, 23], indicating this agent has a broad host range. We detected *A. bovis* in five tick species (*H. qinghaiensis, D. abaensis*, *D. nuttalli*, *I. crenulatus*, and *H. danieli*) from 14 sampling sites and it has the highest infection rate when compared with *A. phagocytophilum*, *A. ovis* and *A. capra*. Five genotypes of *A. bovis* were found, demonstrating its diversity in the ticks of Qinghai. *A. bovis* can be found in many tick species, such as *H. longicornis* in China [24], Korea [25] and Japan [26]. *A. bovis* was also found in *H. lagrangei* in Thailand [27], *H.concinna* in Russia [28], *H. megaspinosa* in Japan [29]; *Amblyomma variegatum* and *R. appendiculatus* in Africa [30], *Rhipicephalus evertsi* in South Africa [31], and *R. turanicus* in Israel [32]. We found *A. bovis* in *H. qinghaiensis, D. abaensis*, *D. nuttalli*, *I. crenulatus*, and *H. danieli* ticks. Statistics analysis indicated that *A. bovi*s was more likely to infect male ticks and ticks at altitude above 4000 m. This result may be related to the distribution of its mammal hosts, since the majority of the yak population lives at altitudes more than 4000 m.

*A. ovis* is widely distributed in Asia, Europe, Africa and North American. Several *msp*4 gene variants of *A. ovis* have been identified in sheep and goats in northwest regions of China [14, 33, 34]. *D. nuttalli*, *Hyalomma asiaticum* and *Rhipicephalus pumilio* are vectors of *A. ovis* in China [9]. We detected *A. ovis* in *D. abaensis*, *D. nuttalli*, *H. tibetensis*, and four *msp*4 gene variants were identified in ticks. These variants showed high similarities to those from Chinese and Spanish strains, indicating diversity of *A. ovis* in the study ticks.

*A. capra* was initially identified in goats, and was subsequently considered to be an emerging human pathogen [1]. *A. capra* was previously identified in *H. qinghaiensis* in Gansu Province, in *H. longicornis* in Shandong Province, and in *I. persulcatus* in Heilongjiang Province [35, 36]. We detected *A. capra* in *H. qinghaiensis, D. abaensis*, and *D. nuttalli*, and two genotypes were identified on the basis of *gltA*, 16S rRNA, *groEL* gene analysis. One genotype showed high sequence identity to the *A. capra* HLJ-14 strain, which had been reported in both goats and humans in China [1]. Another genotype showed low sequence identity to the strain HLJ-14 of *A. capra*, but high identity to an *A. capra-*like bacteria from *H. qinghaiensis* ticks [35]. Additionally, *H. qinghaiensis* is the dominant tick species for the infection of *A. capra*, and high prevalence occurs in the ticks found at altitudes less than 3000 m.

Although the present study has revealed the current status of ixodid tick infestation with *Anaplasma* spp. in the investigated areas, the specific biological vector for the individual *Anaplasma* species need to be further studied by transmission experiments. In addition, the infections of *Anaplasm*a species in animals or humans should be investigated to understand the true impact of anaplasmosis in Qinghai Province.

## Conclusions

We demonstrated the prevalence of *A. bovis*, *A. ovis*, *A. phagocytophilum*, and *A. capra* in ticks from 22 counties of Qinghai Province. *Anaplasma* infection in ticks is associated with the species, gender and distribution of the ticks. The prevalence of *A. capra* in ticks may be a threat to public health in Qinghai Province.

## Additional file

Additional file 1: Multilingual abstracts in the five official working languages of the United Nations. (PDF 726 kb)

## Abbreviations

*gltA*: Citrate synthase
*groEL*: Heat shock protein
