# Supplementary material for: Molecular detection of Anaplasma infections in ixodid ticks from the Qinghai-Tibet Plateau
Source: Infect Dis Poverty. 2019 Feb 7;8:12. doi: 10.1186/s40249-019-0522-z (PMC6366118; doi:10.1186/s40249-019-0522-z)
Supplement: Supplementary file 1 — Multilingual abstracts in the five official working languages of the United Nations. (PDF 726 kb) [file 40249_2019_522_MOESM1_ESM.pdf]

Translation of the abstract into the five official working languages of the United Nations

الكشفالجزيئي عن الإصابة بالأنابلازما في القراد اللبودي بهضبة شنجهاي-التبت

رونج هان، جي-في يانج، محمد عزير مختار، جي تشين، كينج-لي نيو، وان-كينج لين، جوان لي، جان-شون لو، هونج ين، جي-جي لو

ملخّص

الأنابلازما هي فصيلة من البكتيريا الإجبارية داخل الخلايا تنتقل عن طريق القراد و تصيب الكثير من الحيوانات البرية، والمستأنسة، والبشر انتشار بكتيريا الأنابلازما في القراد اللبودي بمقاطعة شنجهاي غير مفهوم بصورة جيدة. تم فحص 1104 من القراد البالغ الباحث عن مضيف إجماليًا في هذه الدراسة للتحقق من إصابتهم ببكتيريا الأنابلازما ونتيجة لهذا، فقد أوضحنا معدلات إصابة إجمالية بنسب 3.1% و 11.1% و5.6% و 4.5%لبكتيريا الأنابلازما (فاجوستوفيلوم) والأنابلازما البقرية، والأنابلازما الغنمية، والأنابلازما الماعزية على التتابع. كانت كل العينات سلبية بالنسبة لـلأنابلازما الهامشية. اختلفت المعدلات الإيجابية للأنابلازما (فاجوسيتوفيلوم) والأنابلازما الغنمية والأنابلازما الماعزية في فصائل القراد المختلفة بنسبة كبيرة. كانت المعدلات الإيجابية للأنابلازما الماعزية، والأنابلازما البقرية أعلى في القراد الذكور عن الإناث بنسبة كبيرة. أظهر تحليل متوالية الأنابلازما الغنمية تطابقًا بنسبة 99.5 - 100% مع المستخلصات المبلغة السابقة. وتطابق تحليل متوالية الأنابلازما (فاجوسيتوفيلوم) بنسبة 100% مع سلالات SHX21, JC3-Ap- 3 181-and ZAM dog من الأغنام، والغزلان المانغولية، والكلاب.تم العثور على نمطين جينيين من الأنابلازما الماعزية استنادًا إلى تحاليل S rRNA16، و جين سينثيز السترات (gltA)، و جين بروتين الصدمة الحرارية (groEL). والخلاصة؛ تواجدت الأنابلازما البقرية، والأنابلازما الغنمية، والأنابلازما (فاجوسيتوفيلوم)، والأنابلازما الماعزية في القراد بمقاطعة شنجهاي. ترتبط الإصابة بالأنابلازما بفصيلة القراد، و جنسها، وتوزيعها. ستساعد هذه البيانات على فهم حالة انتشار الإصابة بالأنابلازما في القراد بهضبة شنجهاي-التبت.

Translated from English version into Arabic by Duaa Elsharkawy, proofread by Amal Imam, through

TRANSLATORS
WITHOUT BORDERS

青藏高原地区硬蜱感染无浆体病原的分子检测

韩蓉，杨吉飞，Muhammad Uzair Mukhtar，陈泽，牛庆丽，林元清，刘光远，罗建勋，殷宏，刘志杰

摘要

无浆体属病原是一类经蜱传播的严格胞内寄生菌，可感染多种野生动物和家畜，并且感染人；关于青海省硬蜱感染无浆体病原的流行情况知之甚少。本调查了采自青海省 22 个地区植被上的 1104 只饥饿成蜱。结果发现在青海省采集的蜱中嗜吞噬细胞无浆体（*Anaplasma phagocytophilum*）的感染率为 3.1%，牛无浆体（*A. bovis*）的感染率为 11.1%，绵羊无浆体（*A. ovis*）的感染率为 5.6%，山羊无浆体（*A. capra*）的感染率为 4.5 %，没有检测到边缘无浆体（*A. marginale*）阳性样品。这些无浆体病原中，嗜吞噬细胞无浆体，绵羊无浆体，山羊无浆体在不同蜱种中的感染阳性率显著不同。山羊无浆体和牛无浆体在雄性蜱中的感染阳性率显著高于雌蜱。通过序列比对分析发现，本研究获得的绵羊无浆体序列与先前报道的序列相似度达 99.5-100%；获得的嗜吞噬细胞无浆体序列跟先前报道的羊中分离到的 Ap-SHX21 株、蒙古羚羊中分离到的 JC3-3 株和狗中分离到的 ZAM dog-181 株完全一致。基于对山羊无浆体的 16S rRNA, *gltA* 和 *groEl* 基因分析，发现青海省存在两种基因型的山羊无浆体病原。研究表明，在青海省的 22 个地区存在牛无浆体、绵羊无浆体、嗜吞噬细胞无浆体和山羊无浆体病原的感染与流行；无浆体病原的感染阳性率与蜱的种类，蜱的性别和蜱的地理分布有关。上述数据将有助我们深入研究青藏高原地区硬蜱感染无浆体病原的情况。

Translated from English version into Chinese by Rong Han

Détection moléculaire des infections à *Anaplasma* des tiques (Ixodidae) du Plateau du Qinghai (Tibet)

Rong Han, Ji-Fei Yang, Muhammad Uzair Mukhtar, Ze Chen, Qing-Li Niu, Yuan-Qing Lin, Guang-Yuan Liu, Jian-Xun Luo, Hong Yin et Zhi-Jie Liu

Abstrait

Les bactéries du genre *Anaplasma* sont des parasites intracellulaires obligés transmis par les tiques, qui infectent de nombreux animaux sauvages et domestiques ainsi que l'espèce humaine. La prévalence des différentes espèces d'*Anaplasma* parmi les tiques (Ixodidae) de la province du Qinghai est mal connue. Dans le cadre de notre étude, nous avons recherché les infections à *Anaplasma* sur un total de 1104 tiques adultes en quête d'hôte. Par conséquent, nous avons ainsi montré que le taux d'infection total par *A. phagocytophilum* était de 3,1 %, par *A. bovis* de 11,1 %, par *A. ovis* de 5,6 % et par *A. capra* de 4,5 %, respectivement. Tous les échantillons de tiques étaient négatifs pour *A. marginale*. Les taux de sujets positifs pour *A. phagocytophilum*, *A. ovis* et *A. capra* étaient significativement différents parmi les différentes espèces de tiques. Le taux de sujets positifs pour *A. capra* et *A. bovis* était significativement plus élevé parmi les tiques mâles que parmi les femelles. Le séquençage d'*A. ovis* a montré une identité à 99,5-100 % avec les isolats précédemment rapportés. Les séquences d'*A. phagocytophilum* étaient identiques à 100 % aux souches Ap-SHX21, JC3-3 et ZAM dog-181, présentes respectivement chez le mouton, la gazelle de Mongolie et les chiens.Deux génotypes d'*A. capra* ont été identifiés par analyse de l'ARNr 16S, du gène de la citrate synthase (*gltA*) et du gène de la protéine de choc thermique (*groEL*). En conclusion, *A. bovis*, *A. ovis*, *A. phagocytophilum* et *A. capra* étaient présentes dans les tiques de la province du Qinghai. L'infection par les espèces du genre *Anaplasma* dépend de l'espèce de tique, de son sexe et de sa distribution. Ces données seront utiles pour comprendre la prévalence des infections à *Anaplasma* parmi les tiques du plateau du Qinghai (Tibet).

Translated from English version into French by Suzanne Assenat, proofread by Emmanuelle Hardan, through

TRANSLATORS WITHOUT BORDERS

Молекулярное обнаружение инфекций, вызываемых *Anaplasma*, у иксодовых клещей из Цинхай -Тибетского плато

Ронг Хан, Джи-Фей Ян, Мухаммед Узэйр Мухтар, Зе Чен, Цин-Ли Ниу, Юань-Цин Лин, Гуан-Юань Лю, Цзянь-Цзянь Сюнь Ло, Хун Инь и Чжи-Цзе Лю

Тезисы

Микроорганизмы рода *Anaplasma* — это передающиеся клещами облигатные внутриклеточные бактерии, которые заражают диких и домашних животных и людей. Распространенность видов *Anaplasma* spp. у иксодовых клещей провинции Цинхай изучена плохо. В этой работе было исследовано в общей сложности 1104 взрослых клеща на предмет заражения видами *Anaplasma*. В результате мы обнаружили общий уровень заражения 3.1%, 11.1%, 5.6% и 4.5% для *A. phagocytophilum*, *A. bovis*, *A. ovis* и *A. capra*, соответственно. Все взятые образцы клещей были отрицательными на *A. marginale*. Положительные показатели для *A. phagocytophilum*, *A. ovis* и *A. capra* значительно отличались у разных видов клещей. Положительные показатели для *A. capra* и *A. bovis* были значительно выше у самцов клещей, чем у самок. Секвенирование *A. ovis* показало 99,5-100% идентичности с ранее зарегистрированными культурами. Последовательности для *A. phagocytophilum* были на 100% идентичны штаммам Ap-SHX21, JC3-3 и ZAM dog-181 от овец, монгольских газелей и собак. Два генотипа *A. capra* были обнаружены на основе анализарибосомальной РНК 16S, гена цитрат-синтазы (*gltA*) и гена белка теплового шока (*groEL*).

В заключение отметим, что в клещах из провинции Цинхай присутствовали *A. bovis*, *A. ovis*, *A. phagocytophilum* и *A. capra*. Инфекция, вызываемая *Anaplasma*, связана с видами клещей, полом и распространением. Эти данные будут полезны для понимания статуса распространенности инфекций, вызываемых *Anaplasma*, у клещей на плато Цинхай-Тибет.

Translated from English version into Russian by Tatiana Karymshakova, proofread by Michael Orlov, through

Detección molecular de infecciones por *Anaplasma* en garrapatas ióxidas de la meseta tibetana de Qinghai

Rong Han, Ji-Fei Yang, Muhammad Uzair Mukhtar, Ze Chen, Qing-Li Niu, Yuan-Qing Lin, Guang-Yuan Liu, Jian-Xun Luo, Hong Yin y Zhi-Jie Liu

Resumen

Las especies de *Anaplasma* son bacterias intracelulares obligadas transmitidas por garrapatas que infectan a muchos animales salvajes y domésticos y a los seres humanos. La prevalencia de *Anaplasma* spp. en garrapatas ióxidas de la provincia de Qinghai es poco conocida. En este estudio, se ha buscado un total de 1.104 garrapatas adultas para investigar las infecciones producidas por especies de *Anaplasma*. Como resultado, demostramos las tasas de infección totales del 3,1 %, 11,1 %, 5,6 % y 4,5 % para *A. phagocytophilum*, *A. bovis*, *A. ovis* y *A. capra*, respectivamente. Todas las muestras de garrapatas fueron negativas para *A. marginale*. Las tasas positivas de *A. phagocytophilum*, *A. ovis* y *A. capra* en diferentes especies de garrapatas fueron significativamente diferentes. Las tasas positivas de *A. capra* y *A. bovis* en las garrapatas macho fueron significativamente más altas que en las garrapatas hembra. El análisis de secuencia de *A. ovis* mostró una identidad del 99,5 al 100 % con respecto a las cepas notificadas anteriormente. Las secuencias de *A. phagocytophilum* tuvo el 100 % de identidad con las cepas Ap-SHX21, JC3-3 y ZAM dog-181 de ovejas, gacelas de Mongolia y perros. Dos genotipos de *A. capra* fueron encontrados basándose en el análisis genético del ARNr 16S, del gen citrato sintasa (*gltA*) y del gen de la proteína de choque térmico (*groEL*). En conclusión, *A. bovis*, *A. ovis*, *A. phagocytophilum* y *A. capra* estuvieron presentes en las garrapatas de la provincia de Qinghai. La infección por *Anaplasma*se asocia con la especie, el sexo y la distribución de las garrapatas. Estos datos serán útiles para comprender el estado de prevalencia de las infecciones por *Anaplasma* en garrapatas de la meseta tibetana de Qinghai.

Translated from English version into Spanish by Lia Sarra Felip, proofread by Mayra León, through
